# Onycholysis an early indicator of thyroid disease

**DOI:** 10.11604/pamj.2019.32.31.17653

**Published:** 2019-01-17

**Authors:** Malumani Malan, Zhe Dai, Wu Jianbo, Song Ji Quan

**Affiliations:** 1 Department of Dermatology and Venereology at Zhongnan Hospital of Wuhan University PR China; 2 Livingstone Central Hospital, Southern Zambia; 3 Department of Endocrinology at Zhongnan Hospital of Wuhan University Hubei, China; 4 Dermatology and Venereology at Zhongnan Hospital of Wuhan University Hubei, China

**Keywords:** Onycholysis, plummer´s nails, thyroid disease, hypothyroidism, hyperthyroidism, Graves´ disease

## Abstract

Onycholysis is also referred to plummer's nails is a dermatological nail disorder characterized by spontaneous distal separation of the nail plate from the free margin and progressively proximally. We discuss a case of the 38-year-old man with onycholysis associated with hyperthyroidism due to Graves' disease. In this case review, we will discuss an association of onycholysis with thyroid disease and its diagnostic prognosis. Any unexplained onycholysis should prompt the clinician to investigate the client for asymptomatic hyperthyroidism.

## Introduction

Ugly nails can be a source of stress for some people especially women despite them not giving any clinical symptomatic problems apart from being 'ugly' cosmetically. Onycholysis is a dermatological nail disorder which runs a chronic course. It is characterized by spontaneous distal separation of the nail plate from the free margin and progressively proximally [1]. Onycholysis is also referred to Plummer's Nails when it occurs in setting of hyperthyroidism [2, 3]. Epidemiologically, onycholysis is not well documented but it affects persons of all races. The pathophysiology of onycholysis broadly may be idiopathic, traumatic, or secondary to nail bed disorders. It is associated with multifactorial causes: systemic conditions like Thyroid diseases (both hypo and hyperthyroidism) [2, 4], psoriasis, [5] drugs-especially anticancer agents like paclitaxel, [6] other chemical agents and in the case of PATEO syndrome [7]. Infective causes have been associated or compounding factors in the progression of onychomycosis like fungal infections [8] and bacterial infections like Pseudomonas aeruginosa, [1] trauma to nail, [9] and it can also be caused by any local problem, such as periungual warts as documented by Fawcett *et al*. Onycholysis depending on the cause is treatable as evidenced by Li *et al* in which they described a case of onychomadesis associated with chemotherapy in a 72-year-old woman [6]. In this case review, will discuss an association of onycholysis with thyroid disease and its diagnostic prognosis.

## Patient and observation

**History:** A 38-year-old West African married man, employed as an information technology specialist on a working workshop to Wuhan, Hubei province, P.R China presents at the out-patient department of Zhongnan Hospital of Wuhan University with long standing complaints of generalized body weakness, severe sweating even in conducive environmental temperatures, occasional palpitations of the heart for over slightly one year. However, these symptoms got worse 2 days prior presentation to our hospital. He denies any history of headache, fever, fainting episodes, joint pains or swellings, cough, chest pains. He had normal urinary and bowel habitus. He gives a positive history of nail changes about 7 years ago prior the about symptoms. The nail changes firstly involved the fingers of the hands and he was treated as fungal nail infections (onychomycoses) for over 6 months with the condition worsening and the nail changes spread to the toes and other fingers. He denies any history of skin rash or use of any lotion or corrosives agents or ingestion of any drugs prior initiation of these nail changes. He is not diabetic, coronary heart disease, hypertensive but he has had history isolated elevated blood pressure, not treated. HIV/AIDS serological test was negative and so was hepatitis B, C and Syphilis. He has no significant past medical history. No known drug or food allergies and he has not been on any medications in the recent months. He does not smoke cigarettes nor drink alcohol and use of any illicit drugs.

**Examination:** An African man of lean-to fair built with BMI of 19.83 kilogram per meter squared. He was oriented in time, place and persons. He was lightly dressed, diaphoretic with mild proptosis of eyes with mild positive lid lag, Mobilus sign was negative. Positive metabolic flap (resting tremulous hands) He was warm to touch, not pale, conjunctival jaundice and no lymphadenopathy. Vitals: BP 128/56mmHg, Temperature 36.4°C, Respirations of 20/minute, he has a normal regular full volume pulse of 73/min.

**Figure 1:**
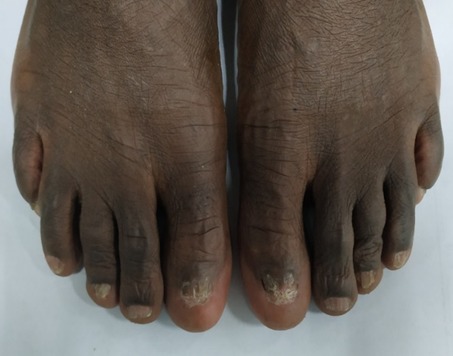
toes showed nail plate thicken, subungual hyperkeratosis, onycholysis especially the big toes

**Figure 2:**
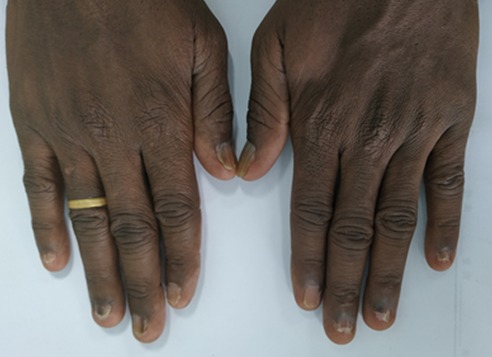
Fingers showing nail plate thicken, subungual hyperkeratosis, onycholysis especially on the left hand involving the little, ring and middle finger and some linear discoloration of nail plates: dark longitudinal streaks on the right ring and middle finger. There was no pitting nor pterygium formation on nails

**Local exam:** Neck; he had a soft nodular goiter, 2^nd^ degree swelling, non-tender and no bruits on auscultation. Skin and Nails; normal skin texture and pigmentation. The nails of the fingers and toes showed nail plate thicken, subungual hyperkeratosis, onycholysis and some linear discoloration of nail plates: dark longitudinal streaks. There was no pitting or pterygium formation Figure 1 and Figure 2. He had no finger clubbing or any joint changes. The rest of the examination was unremarkable.

**Investigations done:** Laboratory tests done from point of care to discharge. The Thyroid profile reviewed raised free T3, T4 and A-TSHR(TRAb) with a suppressed TSH. The full blood count show neutropenia, other tests were unremarkable as indicated in Table 1.

**Table 1:** Laboratory tests done from point of care to discharge

Test	Components	Value in SI Units	Normal Range
Thyroid function tests	Free T3	52.4pmol/L	3.21- 6.5
	Free T4	103.09pmol/L	10.20 – 21.88
	TSH	0.048uIU/ml	0.3 – 4.6
	ATG	12 IU/ml	0.0 -72
	A-TPO	5 IU/ml	0.0 - 16
	A-TSHR(TRAb)	31.34 IU/L	0.0- 1.75
Full Blood Count- WBC	Neutrophils (Ab/%)	1.09 x10^9/L (22.0%)	
	Monocyte (Ab/%)	6.67 x10^9/L (13.7%)	
	Lymphocyte (Ab/%)	3.42x10^9/ L (52.0%)	
RBC	Hemoglobin	128g/L	
	Hematocrit	39.8	
	MCV	78.2fl	
	MCH	25.0pg	
Liver function tests	ALB	37.0g/L	
	Total Protein	60.6g/L	
	ALP	152U/L	

**ATG**-anti-thymocyte globulin, **TSH**-Thyroid stimulating hormone, FT**4**-Free tetraiodothyronine, **FT3**-Free triiodothyronine, **A-TPO**-Thyroid peroxidase antibody, **A-TSHR**-Thyroid stimulating hormone receptor antibody

**Thyroid Emission Computed Tomography (ECT):** The thyroid scan showed diffuse enlargement of both lobes of the thyroid gland with significant enhancement of 99mTc04 in whole gland, which is highly considerable of Hyperthyroidism.

**Electrocardiogram:** Normal sinus rhythm with PR-elongation. The renal functions and cardiac enzymes tests were unremarkable.

**Management:** A multidisplinary approach was followed in the management of patient and we followed the treatment guidelines or protocols for management of endocrine patients in the department. He was managed as a case of thyrotoxicosis in Graves' disease with hyperthyroidism induced onycholysis. He was immediately started on a cocktail of the following drugs: thiamazole tablet 10mg three times a day, Inosine 0.2g three times a day, Leucogenum 20mg three times a day, potassium chloride 0.5g twice daily and recombinant human granulocyte colony-stimulating factor (rhG-CSF) 50µg intravenously every after three days until the neutropenia improves. The client showed much improvement with regards to resolving of the symptoms while in admission and was discharge after 7 days of admission. His condition was explained to him and a detailed treatment plan offered to the patient as he travelled back to his home country-Gambia for continued care.

## Discussion

The skin and its appendages are a window to a range of systemic conditions and as such any cutaneous pathology, prompt consultation to dermatologist should be sort for. Thyroid disease like many other chronic systemic conditions have a constellation of symptoms which range from simple to subtle life threatening. Most clinicians have a biasness to subtle life-threatening conditions as opposed to simple non-problematic condition. For instance, our client had reported to his general practitioner 7 years prior presentation of the other symptoms of thyroid disease. However, the earlier attending clinicians thought it was onychomycoses-nail fungal infection and he was given tablets of Griseofulvin to ingest for over 2 months with the condition worsening and now spreading to other fingers and toes. Onychomycoses has excellent treatment outcome especially with the use of terbinafine. Our client had some linear discoloration of nail plates- Dark longitudinal streaks on the right ring and middle finger, such streaks have been associated with Melanoma, benign nevus, chemical staining and is also a normal variant in darkly pigmented people was the case with our client. It is prudent to also look out for malignancies under the nail plate as they can be the cause of onycholysis. Other causes of onycholysis like Lichen planus, psoriasis, onychomycosis was excluded during careful history taking, physical examination and investigations. The authors are of the view that onycholysis could be an early indication of thyroid disease especially hyperthyroidism as it has been reported in cases of hypothyroidism by Arabatzis *et al.* [10, 11]. Among the symptoms our client had; heat intolerance, and warm, moist skin and hyperhidrosis of the face, palms and soles. These are commonly seen in hyperthyroidism Lause *et al* describes other symptoms like flushing of the face (which is difficult to appreciate in the dark-skinned individuals), erythema of the palms, a downy texture and diffuse scalp hair, soft and shiny nails and pretibial myxedema [12].

The cutaneous features of hyperthyroidism are said to be as a result of Stimulation of the thyrotropin receptor which results in mesenchymal tissue proliferation [12]. As a consequence of the thyrotropin receptor stimulation, the afore mentioned symptoms arise. Like in our client, the TRAb (A-TSHR) was significantly raised about 18 times the upper limit, Kotwal *et al* describes that thyroid stimulating hormone receptor (TSHR) autoantibodies (TRAb) play a central role in the evaluation of Graves' disease and other thyroid disorders of autoimmunity [13]. TRAb by fluoroenzyme immunoassay in latest studies by Villalta *et al* suggest that it may be adopted into clinical practice for the differential diagnosis of hyperthyroidism, to screen for transient hyperthyroidism, and to monitor disease activity and treatment effects-prognosis [14]. In this review, our particular attention is on onycholysis which has a chronic course bearing and of undetermined origin, 'idiopathic'. The author suggests that idiopathic onycholysis could be an early indicator of thyroid disease. The use of broad term as thyroid disease is because both hypo-and hyperthyroidism have been associated with onycholysis. The first ever association case study was recorded in 1958 by Luria *et al.* [15]. In her review the author, Luria *et al.*associated onycholysis of undetermined cause in patients with hyperthyroidism as due to diffuse goiter and records that it has excellent out come if patients' thyroid hormones retains to euthyroid states. Particularly Graves' disease has being noted as the cause of onycholysis associated with hyperthyroidism [16]. Onyiriuka *et al.* documented a case of 16-year-old Nigerian girl with onycholysis associated with Graves' disease [17]. The pathogenesis of hyperthyroidism associated onycholysis is still unknown but from most of the literature search, its observed that any unexplained onycholysis should prompt the clinician to investigate the client for asymptomatic hyperthyroidism [12, 15-17].

## Conclusion

Onycholysis is associated with thyroid disease especially hyperthyroidism. Even if the pathogenesis of this association is still elusive, any client with unexplained onycholysis should be evaluated for asymptomatic thyroid disease and an endocrinologist should be involved soonest. Thus, the author is of the view that a large-scale prospective study should be conducted to validate this causal association between onycholysis and hyperthyroidism, its incidence, pathogenesis and natural course of onycholysis in thyroid disease.
